# The genus *Polystenus* (Hymenoptera: Braconidae: Doryctinae)in China, with descriptions of two new species

**DOI:** 10.1093/jis/14.1.66

**Published:** 2014-01-01

**Authors:** Pu Tang, Sergey Belokobylskij, Xue-xin Chen, Henry Hagedorn

**Affiliations:** 1 State Key Laboratory of Rice Biology, Institute of Insect Sciences, Zhejiang University, Hangzhou 310058, China; 2 Zoological Institute, Russian Academy of Sciences, Universitetskaya nab., 1, St. Petersburg, 199034, Russia; Museum and Institute of Zoology, Polish Academy of Sciences, Wilcza 64, Warszawa 00-679, Poland

**Keywords:** Hecabolini, *Spathiostenus*, taxonomy, photo, key

## Abstract

The species of *Polystenus* Foerster, 1862 (Hymenoptera: Braconidae: Doryctinae) from China are revised, and four species are recognized. Two new species, *P. brevitergum***sp. nov.** and *P. taiwanus***sp. nov.** , are described and illustrated. A key to all species of the genus *Polystenus* is provided.

## Introduction

*Polystenus* Foerster, 1862 (Hymenoptera: Braconidae: Doryctinae) is a small genus distributed in the Palaearctic and Oriental regions. Members are ectoparasitoids of the larvae of wood and bark-boring beetles (Buprestidae, Cerambycidae, and Scolytidae). Until now, four valid species of this genus have been recorded worldwide, namely, *P. rugosus* Foerster, 1862, *P. ruficeps* (Ashmead, 1905), *P. remus* (Nixon, 1943) and *P. ana-colus* ( Chen et Shi, 2004 ) ( Belokobylskij and Maeto 2009 ; Yu et al. 2012 ).

Previous to our study, only two species of *Polystenus* were recorded in China ( Belokobylskij 1996 ; Chen and Shi 2004 ; Belokobylskij and Maeto 2009) . Belokobylskij (1996) recorded *P. rugosus* from Taiwan (material from the American Entomological Institute, Gainesville, USA). The other species, *P. anacolus* , was originally described by Chen and Shi (2004) in the genus *Eucorystoides*Ashmead, 1900 (junior synonym of *Polystenus* ). In this study, four species of this genus were found in China, of which two species are described and illustrated as new species. A key to all species of the genus *Polystenus* Foerster is given.

## Materials and Methods

This study is based on the specimens preserved in the Parasitic Hymenoptera Collection of the Institute of Insect Sciences, Zhejiang University (ZJUH). The type materials of previously described species *Polystenus rugosus* and *P. remus* were studied in the collections of Humboldt University (Berlin, Germany) and the Natural History Museum (London, UK). The terminology and measurements used followed van Achterberg (1993) . Belokobylskij and Maeto (2009) were an additional source for the description of sculpture and setation. Abbreviations used in this paper are as follows: POL = post-ocellar line, Od = ocellar diameter, OOL = oculo- ocellar line. All descriptions and measurements were made under a Leica MZ 12.5 microscope (( www.leicamicrosystems.com ); figures were made by a digital camera (Q-Imaging, Micropublisher, 3.3 RTV, ( www.qimaging.com ) attached to a stereomicroscope (Leica MZ APO) and Auto-Montage Pro version 5.0 software (Syncroscopy, ( www.syncroscopy.com ). Type specimens are deposited in the Parasitic Hymenoptera Collection of the Zhejiang University, Hangzhou, China (ZJUH).

### Nomenclaure

This publication and the nomenclature it contains have been registered in ZooBank. The LSID number is:

http://urn:lsid:zoobank.org:pub:05245476-E6E2-4BF8-9417-D468DF5B78F7

The registration of this paper can be found online by inserting the LSID number after ( www.zoobank.org/

### Taxonomy

***Polystenus* Foerster**

**Diagnosis:** Head transverse. Frons more or less flat and without median keel. Vertex usually completely densely transversely striate. Occipital carina complete dorsally, ventrally oblitereted at short distance upper base of mandible or (rarely) joined ventrally with hypostomal carina. Malar suture absent. Hypoclypeal depression rather small and round. Postgenal bridge narrow, but distinct. Maxillary palpus 6-segmented, labial palpus 4-segmented; third segment of labial palpus long. Scapus rather long and wide, without apical flange and subbasal constriction. First flagellar segment usually weakly longer than second segment. Mesosoma distinctly depressed. Neck of pronotum long, dorsally with high and wide convex lobe (lateral view); pronotal keel absent. Mesonotum (lateral view) weakly and gently-roundly elevated above pronotum. Median lobe of mesonotum without anterolateral corners. Notauli more or less complete, deep anteriorly and very shallow posteriorly, entirely sculptured. Prescutellar depression rather short. Scutoscutellar suture distinct. Scutellum transverse. Precoxal sulcus distinct, long, narrow, more or less straight. Prepectal carina distinct and complete. Postpectal carina absent. Propodeum without areas delineated by carinae or with finely delineated large basolateral areas; lateral tubercles and propodeal bridge absent. Pterostigma of forewing wide; r vein usually arising distinctly behind middle of pterostigma (sometimes form middle). Marginal cell shortened. r-m vein absent. m-cu vein more or less distinctly antefurcal. cua postfurcal. Discal cell shortly petiolate anteriorly. CU1a vein basally distinctly curved. Fisrt subdiscal cell open; CU1b vein absent. In hind wing, cua present. Subbasal cell large; M+CU vein longer than 1-M. m-cu vein present, strongly oblique. Basal cell narrow and short. In male, hind wing without stigma-like enlargement and 1-SC+R vein at least partly absent. Fore tibia with several thick spines arranged in single row. Hind coxa short, subround, without basoventral tooth. Hind femur rather slender, with low dorsal protuberance. First metasomal tergite wide and more or less short; acrosternite not elongate. Dorsope of first tergite distinct; spiracular tubercles weak and situated in basal third of tergite. Second-fifth tergite with distinct Y-shape pale figure. Second suture distinct, complete, with deep lateral curvations. Second tergite with complete convergent furrows delineated U-shape area (as part of Y-shaped figure of metasoma). Third tergite with shallow transverse subbasal furrow.

This genus is closely related to *Spathiostenus* Belokobylskij, but differs in having the acrosternite of first tergite not elongated; first and the following tergites shorter; legs and metasoma of male with short setae; r vein of forewing usually distinctly behind middle of pterostigma (except *P. taiwanus***sp. nov.** )

### Key to species of the genus *Polystenus* Foerster

1. Second and third tergites shagreened. Head (including vertex) smooth. Ovipositor sheath about as long as body. Body length 2.5 mm. Philippines…………… ****P. ruficeps**** (Ashmead) - Second and third tergites striate-reticulate or striate. Head (including usually vertex) sculptured. Ovipositor sheath distinctly shorter than body…………………………………………2

2. Mesoscutum entirely covered by dense, short and semierect pale setae. Ovipositor sheath 1.4 times longer than metasoma. Body length 6.0 mm. China (Taiwan)……………… …………………………. *P. taiwanus***sp. nov.** - Mesoscutum covered by dense, short and semierect pale setae along notauli and laterally, with glabrous median areas on all lobes. Ovipositor sheath not or sometimes weakly longer than metasoma ………………………3

3. First tergite shorter, length almost equal to its apical width. Color of body paler. Mesosoma 2.3 times as long as high. Body length 5.0 mm. China (Henan)……….……………... ……………………… *P. brevitergum***sp. nov.** - First tergite distinctly longer than its apical width. Color of body darker. Mesosoma 3.0– 4.0 times as long as high…...………………..4

4. Ocelli arranged in triangle with base equal to its sides. Ovipositor sheath distinctly shorter than metasoma. Body length 5.9 mm. China (Fujian)…………… ****P. anacolus**** (Chen et Shi) - Ocelli arranged in triangle with base longer than its sides. Ovipositor sheath about as long as metasoma…………………………………5

5. Mesosoma distinctly depressed, its length 3.6–4.0 times maximum height. Setae of hind tibia 0.6–0.7 times maximum width of hind tibia. Acrosternite of first segment not elongated. Metasomal tergites short. Body length 4.8–7.3 mm. China (Henan, Zhejiang, Taiwan (?)), Japan, Korea, Kazakhstan, Tajikistan, Russia (Far East, European part), Europe……. …………………………... ****P. rugosus**** Foerster - Mesosoma less depressed, its length 3.3 times maximum height. Setae of hind tibia 1.00–1.25 times maximum width of hind tibia. Acrosternite of first segment elongated. Metasomal tergites long. Body length 2.5–4.2 mm. India ………………………. ****P. remus**** (Nixon)

*Polystenus brevitergum* **sp. nov.** Holotype. ♀, China, Henan Prov., Neixiang Getiaopa, 12.VII.1998, Chen Xuexin, No. 986013 (ZJUH).

**Description.** Female. Body length 5.0 mm; fore wing length 3.6 mm.

**Head.** Antenna slender, setiform, with more than 33 segments (apical segments missing). Scapus 1.6 times as long as maximum width, almost equal to first flagellar segment. First flagellar segment weakly curved, subcylindrical, 4.2 times as long as its apical width, almost equal to second segment. Head weakly depressed, its width 1.6 times median length, 1.4 times maximum height, equal to width of mesoscutum. Head behind eyes (dorsal view) regularly and distinctly roundly narrowed; transverse diameter of eye 2.2 times longer than temple (dorsal view). Vertex finely, densely and semi-circularly striate, almost smooth posteriorly; frons almost entirely densely and finely striate. Frons more or less flat and without median keel. Temple mostly smooth. Vertex widely glabrous medially, with sparse short and semierect setae laterally and in single transverse median line. Ocelli medium-sized, arranged in triangle with base equal to its sides. POL 0.8 times Od, 0.3 times OOL. Eye glabrous, almost without emargination opposite antennal sockets, 1.1 times as high as broad. Malar suture absent. Malar space 0.2 times height of eye, 0.6 times basal width of mandible. Face convex, its width 0.95 times height of eye and 1.3 times height of face and clypeus combined, densely, rather regularly and weakly curvedly striate, with fine or very fine microsculpture. Clypeus with distinct and rather long lower flange. Occipital carina complete dorsally, not joined ventrally with hypostomal carina being obliterated at wide distance upper base of mandible.

**Mesosoma** . Depressed, its length 2.3 times maximum height. Pronotum (lateral view) long, with strongly convex median lobe dorsally. Mesoscutum (lateral view) very weakly and roundly elevated above pronotum, its median length (dorsal view) 0.9 times maximum width. Mesoscutum densely and distinctly granulate, coarsely rugose and with several undulate striae in wide area on medioposterior 0.6; mesoscutum with dense, short and semierect pale setae only along notauli and laterally. Notauli rather wide, crenulate-rugose. Prescutellar depression shallow, rather wide, with nine carinae, rugulose between carinae, medially 0.4 times as long as scutellum. Scutellum densely and finely granulate, flat, transverse, its maximum width 1.6 times median length. Mesopleuron almost entirely smooth, glabrous medially, with rather long dense and erect setae around it. Subalar depression shallow, rather narrow, coarsely ru- gose-reticulate. Precoxal sulcus distinct, rather shallow, but deep medially, almost straight, smooth, running along almost entire lower part of mesopleuron. Metanotal tooth absent. Metapleuron entirely and coarsely rugose- reticulate. Metapleural lobe small, narrow, rounded apically. Metapleuron glabrous medially, with rather long dense and erect setae around it. Propodeum very weakly roundly slanted (lateral view), without lateral tubercles, spiracle small; without delineated areas, but with fine median and two lateral carinae in basal 0.7, almost entirely densely and small rugose-reticulate.

**Wings.** Forewing 3.6 times as long as maximum width. r vein subperpendicular, arising distinctly behind middle of pterostigma. Marginal cell distinctly shortened. 3-SR : r : 2-SR : 1-SR+M : m-cu = 58 : 8 : 19 : 22 : 13. m-cu vein weakly antefurcal. 1-SR+M weakly sinuate. Discal cell 2.2 times longer than wide; cua straight and weakly inclivous (directed forwards) to M+CU1. M+CU1 vein weakly sinuate. Hind wing, M+CU 0.7 times as long as 1-M. m-cu rather short, antefurcal.

**Legs.** Fore tibia with a few coarse spines arranged in single row. Hind coxa 1.6 times longer than maximum width, almost smooth. Hind femur 4.1 times longer than wide, dorsally very finely striate, mostly smooth. Hind tibia dorsally with long, rather sparse, semierect setae, length of these setae 0.9–1.2 times maximum width of hind tibia; its apex without inner flat setae. Hind tarsus 0.9 times as long as hind tibia. Hind basitarsus slender, without ventral keel, 0.7 times as long as second-fifth segments combined. Second segment of hind tarsus 0.5 times as long as basitarsus, 1.6 times longer than fifth segment (without pretarsus).

**Metasoma.** 1.1 times longer than head and mesosoma combined. First tergite with small spiracular tubercles in basal 0.2; tergite rather weakly and almost linearly widened from base to subapex, weakly narrowed apically. Maximum width of first tergite 1.8 times its minimum width; length almost equal to its apical width, 0.7 times length of propodeum. Median length of second tergite 0.6 times its basal width, 1.1 times length of third tergite. Combined length of second and third tergites 0.8 times their maximum width. Second tergite with complete and convergent posteriorly furrows delineated V-shape area (as part of Y-shaped figure of metasoma). Suture between second and third tergites deep, complete, with two rounded bends situated more closely to middle of tergites then to its sides. First tergite with distinct, widely separated, mostly almost parallel, but convergent posteriorly dorsal carinae, densely and coarsely rugose-reticulate with undulate striation. Second tergite entirely coarsely undulately striate with dense and coarse rugosity between striae. Third-fifth tergites entirely with dense and weakly divergent longitudinal striae and with fine rugulosity between striae. Sixth tergite finely granulate-reticulate, smooth in posterior 0.3. Remaining tergites smooth. Ovipositor sheaths distinctly wide, pointed apically, covered by rather slender dense black setae; sheath length almost equal to metasoma, 1.4 times longer than mesosoma, 0.7 times as long as fore wing.

**Color.** Head brownish yellow. Mesosoma brownish yellow, prescutellar depression, metanotum, and propodeum almost black. Metasoma brownish yellow, first tergite entirely, third–fifth tergites in rather small apical area almost black. Antenna black, two-four basal segments brownish yellow. Palpi yellow. Legs brownish yellow. Ovipositor sheath black. Forewing faintly infuscate. Pterostigma entirely dark brown.

**Male. Unknown.**

**Diagnosis** . This new species is similar to Chinese *P. anacolus* , but differs in having the first tergite shorter (its length almost equal to apical width), ovipositor sheath longer (almost equal to metasoma), ocellar triangle with base distinctly longer than sides, and body color paler. This species also resembles *P. remus* from India, but differs in having the temple short, face high, head weakly depressed, recurrent vein antefurcal (interstitial in holotype of *P. remus* ), mesosoma short and less depressed, neck of prothorax short, acrosternite of first tergite not elongate (elongate in holotype of *P. remus* ), the tergites of metasoma (especially second one) short.

**Distribution** . China (Henan).

**Etymology** . From Latin “brevis” meaning “short”, and “tergum” meaning “tergite”.

*Polystenus rugosus* **Foerster, 1862**

*Polystenus rugosus* Foerster, 1862 : 237; Shenefelt and Marsh, 1976 : 1361; Papp, 1984 : 182; Belokobylskij and Tobias , 1986: 34; Belokobylskij, 1996 : 172; 1998: 74; Belokobylskij and Maeto, 2006 : 738, 2009: 409.

*Corystes aciculatus* Reinhard, 1865 : 259. *Eucorystes aciculatus* : Marshall, 1888 : 204, 206.

*Eucorystoides aciculatus* : Ashmead, 1900 : 368; Shenefelt and Marsh, 1976 : 1354; Papp, 1984 : 182 (as syn. of *P. rugosus* ).

**Material examined** . 1♀, China, Henan Prov., Neixiang Baotianman, 15.VII.1998, 1800 m, Ma Yun, No. 987176; 1♀, China, Zhejiang Prov., Hangzhou, 28.VI.1991, Gao Qikang, No. 911394 (ZJUH).

**Distribution** . China (Henan, Zhejiang, Taiwan (?)); Japan, Korea, Kazakhstan, Tajikistan, Russia, Belorussia, Ukraine, Western and Central Europe.

*Polystenus taiwanus* **sp. nov.**

Holotype. ♀, China, Taiwan Prov., Hengchun Redaishiyanzhongxin, 7.XI.2010, Chen Xuexin, No. 201105605 (ZJUH).

**Description** . Female. Body length 6.0 mm; fore wing length 3.9 mm.

**Head.** Antennae rather slender, almost filiform, more than 34-segmented (apical segments missing). Scapus 1.6 times longer than its maximum width, 0.75 times as long as first flagellar segment. First flagellar segment weakly curved, subcylindrical, 5.3 times longer than apical width, 0.9 times as long as second segment. Head weakly depressed, its width 1.6 times median length, 1.4 times maximum height, 1.1 times width of mesoscutum. Head behind eyes (dorsal view) regularly and distinctly roundly narrowed; transverse diameter of eye 2.0 times longer than temple (dorsal view). Vertex at least in anterior 0.7 finely, densely and semi-circularly striate, almost smooth posteriorly; frons almost entirely densely and finely striate. Frons more or less flat and without median keel. Temple mostly smooth. Vertex widely glabrous medially, with sparse, short and semierect setae laterally. Ocelli medium-sized, arranged in triangle with base 1.4 times its sides. POL 1.4 times Od, 0.5 times OOL. Eye glabrous, almost without emargination opposite antennal sockets, 1.3 times as high as broad. Malar suture absent. Malar space 0.3 times height of eye, 0.7 times basal width of mandible. Face convex, its width 0.9 times height of eye and 1.2 times height of face and clypeus combined, densely, rather regularly and weakly curvedly striate, with fine or very fine additional microsculpture. Clypeus with distinct and rather long lower flange. Occipital carina complete dorsally, not joined ventrally with hypostomal carina being obliterated at rather short distance upper base of mandible.

**Mesosoma.** Distinctly depressed, its length 3.0 times maximum height. Pronotum (lateral view) long, with strongly convex median lobe dorsally, with rather distinct pronotal keel situated in its middle. Mesoscutum (lateral view) very weakly and roundly elevated above pronotum, its median length (dorsal view) almost equal to maximum width. Mesoscutum densely and distinctly granulate, laterally with additional rugulosity, coarsely rugose and with several undulate striae in wide area on medioposterior 0.7; mesoscutum entirely with dense, short and semierect pale setae. Notauli rather wide, crenulate-rugose. Prescutellar depression shallow, rather wide, with five carinae, rugulose between carinae, medially 0.4 times as long as scutellum. Scutellum sparsely punctate, flat, transverse, its maximum width 1.2 times median length. Mesopleuron almost entirely smooth, glabrous medially, with rather long dense and erect setae around it. Subalar depression shallow, rather narrow, coarsely rugose-reticulate. Precoxal sulcus distinct, rather shallow, but deep medially, almost straight, smooth, running along almost entire lower part of mesopleuron. Metanotal tooth very short. Metapleuron entirely and coarsely rugose-reticulate. Metapleural lobe small, narrow, rounded apically. Metapleuron mostly glabrous, with rather long dense and erect setae ventrally. Propodeum very weakly roundly slanted (lateral view), without lateral tubercles, spiracle small; without delineated areas, with fine median carina in basal 0.7, almost entirely densely rugose- reticulate.

**Wings.** Forewing 4.4 times longer than its maximum width. r vein subperpendicular, arising almost from middle of pterostigma. Marginal cell distinctly shortened. 3-SR : r : 2-SR : 1-SR+M : m-cu = 67 : 7 : 17 : 22 : 12. m-cu vein distinctly antefurcal. 1-SR+M weakly sinuate. Discal cell 3.0 times longer than wide; cua straight and weakly inclivous (directed forwards) to M+CU1. M+CU1 weakly sinuate. Hind wing, M+CU 0.5 times as long as 1-M. m-cu rather short, very strongly desclerotized, antefurcal.

**Legs.** Fore tibia with a few coarse spines arranged in single row. Hind coxa 1.7 times longer than maximum width, almost smooth. Hind femur 4.4 times longer than wide, dorsally very finely striate, smooth at most part. Hind tibia dorsally with long, rather sparse, semierect setae, length of these setae 0.9–1.5 times maximum width of hind tibia; its apex without inner flat setae. Hind tarsus 0.9 times longer than hind tibia. Hind basitarsus slender, without ventral keel, 0.7 times as long as second–fifth segments combined. Second segment of hind tarsus 0.5 times as long as basitarsus, 1.6 times longer than fifth segment (without pretarsus).

**Metasoma.** 1.2 times longer than head and mesosoma combined. First tergite with small spiracular tubercles in basal 0.3; tergite rather weakly and almost linearly widened from base to subapex, weakly narrowed apically. Maximum width of first tergite about 2.0 times its minimum width; length 1.7 times its apical width, almost equal to length of propodeum. Median length of second tergite equal to its basal width, 1.05 times length of third tergite. Combined length of second and third tergites 1.3 times their maximum width. Second tergite with complete and convergent posteriorly furrows delineated V-shape area (as part of Y-shaped figure of metasoma). Suture between second and third tergites deep, complete, with rounded bends situated more closely to middle of tergites then to its sides. First tergite with distinct, widely separated, almost parallel mostly and convergent posteriorly dorsal carinae, densely and coarsely rugose-reticulate with undulating striation. Second tergite entirely coarsely undulately striate with dense and coarse rugosity between striae. Third–fifth tergites entirely with dense and weakly divergent longitudinal striae and with fine ground rugulosity between striae. Sixth tergite finely granulate-reticulate, smooth in posterior 0.3. Remaining tergites smooth. Ovipositor sheaths distinctly widened, pointed apically, covered by rather slender dense black setae, 1.4 times as long as metasoma, 2.3 times longer than mesosoma, 1.2 times as long as forewing.

**Color.** Head brownish yellow, with reddish- brown tint dorsally. Mesosoma almost black, mesoscutum black with brownish yellow spot, lower part of propleuron brownish yellow. First tergite entirely black, second tergite widely medially, third–six tergites in rather wide area almost black, the rest part brownish yellow. Antenna black, two-four basal segments brownish yellow. Palpi yellow. Legs brownish yellow, hind femur and tibia infuscate. Ovipositor sheath black. Forewing faintly infuscate. Pterostigma dark brown, faintly paler apically.

**Male. Unknown.**

**Diagnosis** . This new species is similar to *P. rugosus* , but differs in mesoscutum entirely with dense and short setae; ovipositor sheath distinctly longer than metasoma; length of first tergite longer (1.7 times its apical width), and vein r arising almost from middle of pterostigma. This new species is closely related to the genus *Spathiostenus* Belokobylskij, but differs in acrosternite not elongate, about 0.25 times as long as first tergite, and first and following tergites shorter.

**Distribution** . China (Taiwan).

**Etymology** . From the Taiwan Island, the type locality of the species.

**Figures 1–8.**
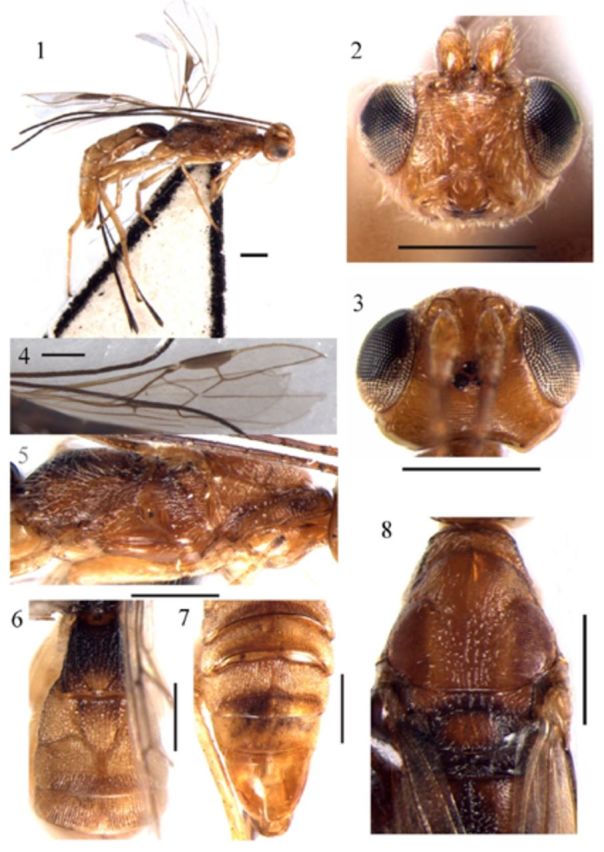
*Polystenus brevitergum* **sp. nov. 1** , habitus, lateral aspect; **2** , head, front aspect; **3** , head, dorsal aspect; **4** , forewing; **5** , mesosoma, lateral aspect; **6** , first–third tergites of metasoma, dorsal aspect; **7** , fourth–seventh tergites of metasoma, dorsal aspect; **8** , mesosoma, dorsal aspect. Scale bar = 0.5 mm. High quality figures are available online.

**Figures 9–17.**
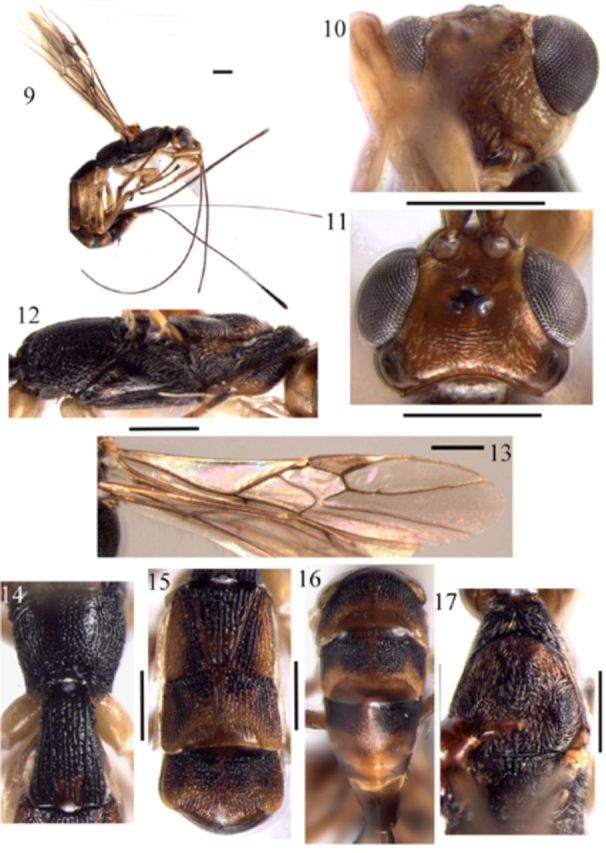
*Polystenus taiwanus* **sp. nov. 9** , habitus, lateral aspect; **10** , head, front aspect; **11** , head, dorsal aspect; **12** , mesosoma, lateral aspect; **13** , wings; **14** , propodeum and first tergite, dorsal aspect; **15** , second–fourth tergites of metasoma, dorsal aspect; **16** , fifth–seventh tergites of metasoma, dorsal aspect; **17** , mesonotum, dorsal aspect. Scale bar = 0.5 mm. High quality figures are available
